# The Effect of Copayments for Prescriptions on Adherence to Prescription Medicines in Publicly Insured Populations; A Systematic Review and Meta-Analysis

**DOI:** 10.1371/journal.pone.0064914

**Published:** 2013-05-28

**Authors:** Sarah-Jo Sinnott, Claire Buckley, David O′Riordan, Colin Bradley, Helen Whelton

**Affiliations:** 1 Department of Epidemiology and Public Health, University College Cork, Cork, Ireland; 2 Department of General Practice, University College Cork, Cork, Ireland; 3 Oral Health Services Research Centre, University College Cork Dental School, Wilton, Cork, Ireland; Groningen Research Institute of Pharmacy, United States of America

## Abstract

**Introduction:**

Copayments are intended to decrease third party expenditure on pharmaceuticals, particularly those regarded as less essential. However, copayments are associated with decreased use of all medicines. Publicly insured populations encompass some vulnerable patient groups such as older individuals and low income groups, who may be especially susceptible to medication non-adherence when required to pay. Non-adherence has potential consequences of increased morbidity and costs elsewhere in the system.

**Objective:**

To quantify the risk of non-adherence to prescribed medicines in publicly insured populations exposed to copayments.

**Methods:**

The population of interest consisted of cohorts who received public health insurance. The intervention was the introduction of, or an increase, in copayment. The outcome was non-adherence to medications, evaluated using objective measures. Eight electronic databases and the grey literature were systematically searched for relevant articles, along with hand searches of references in review articles and the included studies. Studies were quality appraised using modified EPOC and EHPPH checklists. A random effects model was used to generate the meta-analysis in RevMan v5.1. Statistical heterogeneity was assessed using the I^2^ test; p>0.1 indicated a lack of heterogeneity.

**Results:**

Seven out of 41 studies met the inclusion criteria. Five studies contributed more than 1 result to the meta-analysis. The meta-analysis included 199, 996 people overall; 74, 236 people in the copayment group and 125,760 people in the non-copayment group. Average age was 71.75years. In the copayment group, (verses the non-copayment group), the odds ratio for non-adherence was 1.11 (95% CI 1.09–1.14; P = <0.00001). An acceptable level of heterogeneity at I^2^ = 7%, (p = 0.37) was observed.

**Conclusion:**

This meta-analysis showed an 11% increased odds of non-adherence to medicines in publicly insured populations where copayments for medicines are necessary. Policy-makers should be wary of potential negative clinical outcomes resulting from non-adherence, and also possible knock-on economic repercussions.

## Introduction

In the last decade spending on pharmaceuticals in OECD countries has risen by 50% [1]. This has led to increased financial pressures in health systems and many countries have attempted to scale back public expenditure on pharmaceuticals; the US, Canada, Australia, Ireland and South Korea have introduced copayment policies to offset growing drug bills [2]–[7]. A copayment is a fixed fee for a prescription. In theory, copayments are intended to reduce drug expenditure by reducing moral hazard associated with medicines supplied at reduced or zero cost. That is, copayments dis-incentivise the collection of medicines that patients do not consume at home or which have no role in improving health – thus reducing waste [8]. A further function of copayments is to generate revenue to offset drug budget costs. The success of copayment policies, however, depends on the ability of patients to make rational choices about which medications they should or should not take [9]–[14]. Copayments may be disadvantageous if they cause a decrease in use of medications that are beneficial to health.

The impact of copayment policies in different countries has been assessed in various ways, with significant differences in populations studied, methodologies employed and outcome measures described.

Vulnerable populations are those who have increased sensitivity to adverse health outcomes and typically include older people and those on low incomes [15]. Patient groups such as these are commonly covered by public insurance schemes such as Medicaid and Medicare in America, or the General Medical Services scheme in Ireland. Therefore, publicly insured populations may provide a proxy for identifying vulnerable populations.

Assessing the effects of copayments on adherence to prescribed medications in specific populations may offer practical insights, rather than studying general populations; where effect sizes may be diluted [16]. Previous reviews have suggested that patients with low income and chronic disease are particularly susceptible to the unfavourable effects of copayments [17] and that older patients reduce their use of medications in the presence of copayments [16]. Contrary to this, another review [18] stated that poorer and older people may be less sensitive to prescription fees than other reviews had previously reported [16], [19]. A reason for this contrast may be differing included studies in reviews, with associated differences in heterogeneity amongst interventions, outcomes and study designs. A review carried out by Rice *et al* [20] showed that copayments are associated with a decline in health status of older patient groups, with two notable exceptions; those with serious health conditions and those on lower incomes who receive a “financial cushion” around copayments. This evidence, though, is limited by the methodological shortcomings of included studies, including cross-sectional and self-reported data. Furthermore, the outcome of interest in included studies varied and contained patient expenditure, health outcomes and drug utilization.

“Utilization” is an umbrella term which includes the supply, prescription, and use of medicines in a society, with attention to the resulting medical, social, and economic consequences [21]. A more specific outcome than utilization is adherence, which is a component of utilization and refers specifically to “*the extent to which patients take their medicines as prescribed*” [22]. Reviews in the past have focused on utilization; however, the effect of copayments on adherence is increasingly being researched. It is generally accepted that reduced adherence, which may occur in response to a copayment, leads to poorer health outcomes and increased costs for a health service through hospital admissions and hospital care [23]–[29]. Furthermore, improved adherence can lead to savings in health expenditures [30], [31].

One review has focused on the effects of patient cost sharing on adherence to medicines in a general population [32]. This review and other similar reviews which studied utilization as the main outcome, have quantified the effects of copayments on utilization/adherence by estimating price elasticities of demand [16], [18], [33], [34]. Price elasticites of demand indicate how responsive demand is to price. Variable elasticities are noted across these reviews, ranging from 2% to 8% in a general population. Not all reviews categorized their findings by specific population subgroups [32], [33] and none use a homogenous outcome measure. Due to the heterogeneity of included studies in these reviews, it may be possible that summary elasticities do not reflect the true picture, given that it may not have been appropriate to combine individual study effects. Despite numerical differences in elasticities, the direction of results is agreed upon by a Cochrane review in the broad area of cost-sharing, which used the literature published up until 2007. This review echoes the general findings of other reviews; a decreased use of all medicines albeit with a greater decrease in non-essential medicines [35]. An essential medicine is one which is said to proffer health benefits in disease and prolong life, while a non-essential drug is useful in alleviating symptoms only.

Because of inconsistencies in previous reviews and the lack of a meaningful quantitative summary effect of copayments on adherence; this review aimed to consider and quantitatively summarise comparative studies which used an objective measure of adherence. Publicly insured populations typically comprise older and low income individuals, thus the effect in this population was sought as a proxy for identifying vulnerable populations. To date, no review has focused on publicly insured populations. It was hoped that objective measures of adherence, namely Proportion of Days Covered (PDC) and the ReComp Algorithm [36]–[40] would reduce the heterogeneity of evidence examined. Thus, the question this review seeks to answer is “How do copayments affect adherence to prescribed medications in publicly insured populations?”

## Methods

A recent Cochrane review [35] informed the selection of search terms for this review. However, as the Cochrane review focused on utilisation, some modifications were made to encompass terms that would capture studies examining adherence. Study type filters were amended to include cohort studies (**Table 1**). Eight databases were searched including; PubMed, Medline(Ovid), Cinahl, EMBASE, EconLit, SCOPUS, Web of Knowledge and the Cochrane Library. The grey literature was also searched through the WHO, OECD and SIGLE. The references of eligible published studies were hand-searched, as were the references of previously published systematic reviews [18], [32]–[35]. There were no language restrictions on searches and the date range extended from 1946 to 2012. Searches were carried out between November 2011 and December 2011. Searches were updated in September 2012. For a study to be eligible for inclusion in the review the following criteria were required: First, the participants received healthcare from a public insurance scheme. The comparator group was the same population/similar population who either didn't pay copayments or experienced no increase in copayment. Second, the intervention was copayment; either an increase in an existing copayment or the introduction of a copayment. Other types of cost-sharing, for example co-insurance, were excluded. Third, the outcome measure was non-adherence. Four commonly used objective measures of adherence were included in search terms; Proportion of Days Covered (PDC), Medication Possession Ratio (MPR), Daily Defined Dose (DDD) and the ReComp algorithm [36], [37], [40]. Although the DDD is generally a measure of utilisation, it was included in this search as a conservative approach to capturing appropriate studies as some studies reporting DDDs may have included indications of adherence. Non-adherence is classified as any percentage of adherence <80%, an arbitrary but accepted cut-off [36], [41]. Next, types of studies included were randomised controlled trials, controlled before and after studies, interrupted time series designs, repeated measures designs, and cohort designs. The types of studies involved were drawn from, and built upon, the study designs used by the Cochrane Effective Practice and Organisation of Care (EPOC) Review group. Lastly, only adjusted estimates of adherence were included in the meta-analysis [42].

**Table 1:** Search terms used in searches of electronic databases.

Intervention	Outcome	Study Filters
Cost sharingDeductibles and coinsurance*Capitation feeFees, pharmaceuticalFees and charges	Medication adherencePatient compliancePharmaceutical preparation*Prescription drugsDrug costsDrug Utilization*Drug prescriptions	Randomized controlled trial (publication type)Controlled clinical trial (publication type)Intervention studiesEvaluation studies (publication type)Comparative studies (publication type)Retrospective cohort
TextwordsCost shar*Co-paymentCo-pay*CopaymentCopay*Co paymentCo pay*	TextwordsMedication possession ratioDefined Daily DoseProportion Days CoveredReCOMP algorithm	TextwordsExperiment*Time seriesInterrupted time series(Pre test or pretest or (posttest or post test)ImpactIntervention*Effect*Evaluat*
		NOTLetterCommentEditorial

These search terms or variants were used in all databases

Searches were carried out by the main reviewer (SJS). Exclusion of titles and abstracts were confirmed with DOR and CB. Authors of any relevant abstracts which did not have a retrievable whole paper were contacted via email for follow up on subsequent publishing of whole papers. Data extraction was carried by SJS and duplicated by DOR and CB using standardised data extraction forms. Data extracted included general demographic information, copayment value, copayment status, outcome measure used, follow up time and adjusted odds ratios. Disagreements were resolved by discussion and where necessary involved other authors (CBr and HW). When required, authors were contacted by email for data. If no response was gained, a reminder email was sent. The study was excluded if there was no response. Controlled before and after studies and interrupted time series designs were assessed for quality and risk of bias using a modified version of the EPOC Data Collection Checklist and Quality Criteria for CBA and ITS [43]. Using this tool, studies could be rated as strong, moderate, weak or fatally flawed. A study was rated as “weak” if two or more criteria were unmet. Cohort studies were assessed for quality and risk of bias using the Effective Public Health Practice Project component rating scale [44]. Using this tool, studies could be given strong, moderate or weak status. A study was rated as “weak” if it was given two weak ratings across the constituent criteria. Both tools for quality included an assessment of confounding.

RevMan version 5.1 [45] was used to carry out the meta-analysis. The log (OR) and corresponding standard error were inputted. The summary effect measure calculated was the odds ratio and its corresponding 95% confidence interval. Statistical heterogeneity was assessed using the I^2^ test for heterogeneity in RevMan. A conservative value of p>0.1 indicated a lack of heterogeneity. A random effects model was used. The outcomes of all PDC and ReCOMP studies were combined in one meta-analysis because they both measure the same outcome; adherence. A sensitivity analysis was performed to ensure suitability of combination – the combination of the two measures did not distort the conclusion. Sensitivity analyses for publication types and demographics (gender) were also run, no differences in conclusion were observed (Figures S1, S2, S3, S4). The medicines that occurred in the included studies are medicines used in chronic disease and have been referred to as being essential [35]. Therefore, a degree of homogeneity across these medicines permits combination in one meta-analysis.

Publication bias was assessed using visual inspection of a funnel plot generated in RevMan. Formal tests of asymmetry were not appropriate due to similar numbers involved in studies and the lack of more than ten studies in the meta-analysis. The small study effect was investigated by sensitivity analysis and fixed model random effects model comparisons.

## Results

### Search results and study characteristics

**Figure 1:**
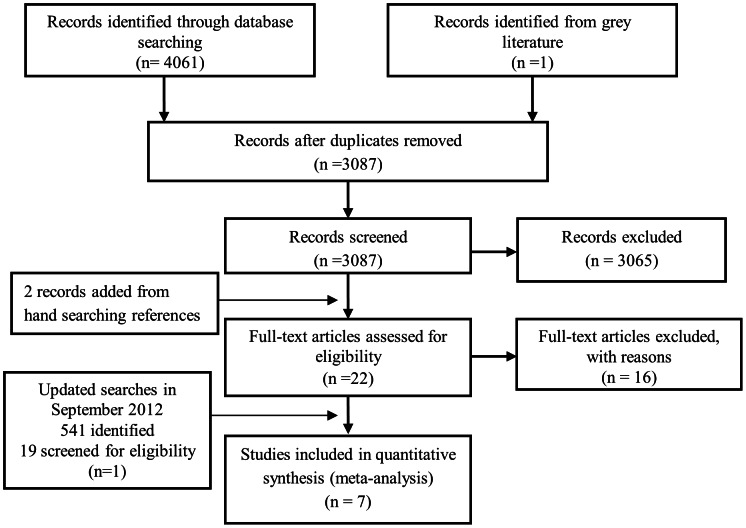
A PRISMA flowchart outlining the procurement of 7 included studies.

From the initial searches, 6 out of 22 studies met the inclusion criteria for meta-analysis [46]–[51]. An additional study [52] out of 19 studies was added from a search update in September 2012 to give 7 included studies overall. A Prisma flowchart (**Figure 1**) demonstrates how the search results were obtained and sequentially ruled out from final inclusion. **Table 2** gives details of the 7 studies included in the meta-analysis. All studies were carried out in the US, despite no geographical limitations in search. Four studies focused on Medicare insurance plans [46], [47], [51], [52] and 3 studies analysed copayment increases in Veteran Affairs [48]–[50]. The average age of patients included in studies was 71.75years (range 64.8yrs to 85+yrs). Gender was evenly distributed between copay and non-copay groups except in Veteran studies which were predominantly male. Five studies [46]–[48], [51], [52] examined adherence using the PDC measure and 2 studies [49], [50] used the ReComp measure of adherence. Four studies were cohort designs and 3 studies were controlled before and after studies. The meta-analysis includes 7 studies in 16 separate patient/medication groups, because 5 studies analysed adherence to medication groups individually or analysed patient groups at different levels of morbidity [46], [49]–[52]. The value of copayments ranged from $5 to $70. Details of excluded studies are included in Table S1.

**Table 2:** Description of studies that examined the effect of a requirement to pay a copayment for prescription drugs on adherence as measured by PDC or ReComp algorithm.

	Setting	Sample Size and Characteristics	Type of study	Adherence measured by	Intervention	Follow up	Result
Doshi et al, 2009 [48]	Veterans in Philadelphia and 7 surrounding counties in Delaware, New Jersey and Pennsylvania who take lipid lowering medicines	2811 Veterans. Average age in copayment group was 76.5 yrs and 70.1 yrs in control group. 99% male. A large proportion are African-American.	Controlled before after	PDC	Increased copayment from $2–$7	24 months post intervention	Increasing non-adherence when subjected to copayment OR 1.63 CI (1.25–2.13)
Polinski et al, 2011 [51]	Medicare Part D beneficiaries in stand alone part D plans, aged 65 years or older. Medications studied included those used to treat: rheumatoid arthritis, CV disease, diabetes, depression or dementia. Data derived from a national dataset.	315703 people between both early and late stages of study. This number might actually be smaller as the same people may be in both early and late cohorts. Average age was 75 yrs. Sample was 63% female. Sample was 87.5% white, 8.5% black and 4% other race.	Cohort study	PDC	Increased copayment to 100% of drug cost when in donut hole for those who were “exposed”.	3.6 months on average.	Increasing non-adherence when subjected to copayment* Any drug OR 1.3 CI (1.25–1.36) CV drug OR 1.23 CI (1.18–1.27) Oral hypoglycaemic drug OR 1.09 CI(1.01–1.18)
Gu et al, 2010 [47]	Patients over 65 yrs and continuously enrolled in Medicare Part D with at least 2 diabetes prescriptions per year. Data derived from a national American dataset.	9521 people. Mean age 75 yrs. 48.4% male. Majority of sample was from Midwest.	Controlled before and after study.	PDC	Increased copay when patients without coverage reached the donut hole. Prices could go from $18.63 to $69.20.	Study period is 1 yr, however unsure of how long patients are followed for in donut hole.	Increasing non-adherence when subjected to copayment OR 1.21 CI (1.07–1.37)
Fung et al, 2010 [46]	Californian over 65 s enrolled in Medicare Part D who were prescribed oral diabetes, hypertension and hyperlipidaemia medicines.	7059 people. 58% were between 65–74 yrs. 48% female (whole sample including those who didn't reach donut hole).	Cohort Study	PDC	Increased copayment when patients without coverage reached the donut hole. Original copayments varied from $5–30 for generic drugs and $10–75 for brand name drugs for a 100 day supply. No information is given on copayment increase.	3 months	Increasing non-adherence when subjected to copayment Hypertension OR 1.28 CI (1.2–1.35) Diabetes OR 1.2 CI (1.14–1.26) Lipid Lowering OR 1.45 CI (1.36–1.54)
Li et al, 2012 [52]	Nationally representative sample of Medicare beneficiaries with diagnoses of hypertension and hyperlipidaemia. Medication classes reported on; anti-hypertensives, lipid lowering drugs, acid suppressants, pain relievers and anti-depressants.	83921 people. Mean age 76.2 years. 28% male. 74.86% white, 11.98% black and 12.66% other ethnicity.	Controlled before and after study	PDC	Increased copayment when patients without coverage reached the donut hole	6 months	Increasing non-adherence when subjected to increased copayment in donut hole. Hypertension OR 1.6 CI (1.5–1.71) Lipid Lowering OR 1.59 CI (1.5–1.68)
Wang et al, 2011 [49]	Veterans at 4 American study sites (locations unknown) who are classified as either low morbidity or high morbidity and are prescribed medicines for hypertension or diabetes.	In the diabetes low morbidity group: 1660 people. Mean age 66.4 yrs. 99% male. 54% white with 38.9% race unknown. In the diabetes high morbidity group: 478 people. Mean age 66.8 yrs. 98% male. 66% white race and 22% unknown race. In the hypertension low morbidity group: 6039 people. Mean age 67.8 yrs. 97.5% Male. 49.6% White race and 43.3% race unknown. In the hypertension high morbidity group: 1051 people. Mean age 67.5 yrs. 98.3% Male. 71.3% white race and 10.9% race unknown (range from 1.5% to 18.5%).	Retrospective Cohort study	ReCOMP algorithm	Increased copayment from $2–$7 *also a copayment introduced two months prior to this affecting primary care visits and speciality care visits. The authors made attempts to control for this policy change.	23 months (have split follow up into a 12 month proximal postperiod and an additional 11 months after this to make up the long period follow up.)	Increasing non-adherence when subjected to copayment. Hypertension Low Morbidity OR 1.19 CI (1.101–1.2789) Hypertesnion High MorbidityOR 1.44 CI (1.20–1.71) Diabetes Low Morbidity OR 1.38 CI (1.191–1.611) Diabetes High Morbidity OR 1.66 CI (1.25–2.21)
Maciejewski et al, 2010 [50]	Veterans at 4 American study sites (locations unknown) who have been diagnosed with hypertension or diabetes and veterans who have hypertension or diabetes and are prescribed statins.	In the diabetes group: 2138 people. Mean age 65.5 yrs. 98.9% male. 56.3% White race and 35% unknown race. In the hypertension group: 7090 people. Mean age 66.8 yrs. 97.7% male. 52.8% white race and 39.1% unknown race. In the statin group: 4048 people. Mean age 67.2 yrs. 99.2% Male. 54.3% white race and 40% race unknown.	Retrospective Cohort study	ReCOMP algorithm	Increased copayment from $2–$7 *also a copayment introduced two months prior to this affecting primary care visits and speciality care visits. The authors made attempts to control for this policy change	23 months (have split follow up into a 12 month proximal postperiod and an additional 11 months after this to make up the long period follow up.)	Increasing non-adherence when subjected to copayment in diabetes and hypertension analyses. Diabetes OR 1.48 CI (1.23–1.79) Hypertension OR 1.13 CI (1.029–1.2481) Lipid Lowering OR 1 CI (0.8522–1.17339)

* Results obtained from contact with authors

### Quality assessment

**Table 3** gives the details of quality assessment of the studies. Included studies were all of weak or weak-moderate strength. Sources of weakness were derived from characteristics imbalances in copayment groups and non-copayment groups, along with poor information given on follow up/attrition.

**Table 3:** Quality assessment of included studies, includes cohort studies and controlled before and after studies.

Cohort Studies									
	Selection Bias	Allocation Bias	Confounding	Blinding	Data Collection Objective	Attrition Bias	Intervention Integrity	Statistics	Overall Strength
Fung et al, 2010 [46]	Unclear – could be a health worker effect present – the non copayment group derives coverage from employers whereas the copayment group must obtain coverage individually.	No	Propensity score matched. Ethnicity not included – sensitivity analysis ruled out importance	Not mentioned	Yes	Unclear	Yes	Yes	Weak
Polinski et al, 2011[51]	Unexposed group is a composite group made of three separate groups. Retirees appear to use more medicines than exposed groups. There were some differences in age, gender and ethnicity.	No	Propensity matched on a wide range of variables. Authors split study into an early and established group because there was no baseline data for the early group.	Not mentioned	Yes	No	Yes	Yes	Weak
Wang et al, 2011 [49]	No, although may be education differences between groups 1 and 8. See Doshi.	No	Propensity matched, but some differences in groups prevailed. Income/neighbourhood proxy for income not included.	Not Mentioned	Yes	Unclear	Yes	Yes	Weak
Maciejewski et al, 2010 [50]	No – see Doshi and Wang	No	Propensity matched, but some differences in groups prevailed Income/neighbourhood proxy for income not included.	Not mentioned	Yes	Unclear	Yes	Yes	Weak

Controlled before and after studies									
	Baseline Measurement	Characteristics for studies using second site as control	Confounding	Blinded assessment of primary outcome	Protection against contamination	Reliable primary outcome measure	Other Risks of Bias	Follow up of patient/attrition	Overall Strength
Doshi et al, 2009 [48]	Met	Met	Intervention that occurred prior to this not controlled for. Household income could be an important confounder but is controlled for in regression.Copayment group- more morbid – adjusted for. Adherence falling in all groups – possibly unknown factor in background. No explanation.	Unclear	Met	Met	Priority group 1is defined on service connected disability – perhaps the people in these groups were involved in frontline services whereas those in priority 8 may have been more senior/more educate. Groups may have been different in this regard and is not controlled for.	Met	Weak
Gu et al, 2010 [47]	Met	Met	An important difference between groups -the copayment group uses more insulin than the non-copayment group – indicating a more morbid/sicker group Regression analysis used.	Unclear	Met	Met	Authors say they control for self –selection into diff insurance policies by using a difference in difference analysis. Not clear how this controls for selection bias.	Unclear (no information on attrition or average follow up length in gap)	Weak
Li et al, 2012 [52]	Met	Met	Substantial difference between intervention and comparison group e.g., ethnicity, morbidity, education.	Unclear	Met	Met	Met	Met	Weak-Moderate

### Publication bias

Asymmetry was noted in the funnel plot. Asymmetry may be due to a publication bias, that is, authors do not publish studies of no effect, which results in an overestimation of the true effect. However, asymmetry may also be caused by weak methodological practices in studies. In this meta-analysis all included studies were of weak design. The small study effect was not present.

### Non-adherence when exposed to copayment

**Figure 2:**
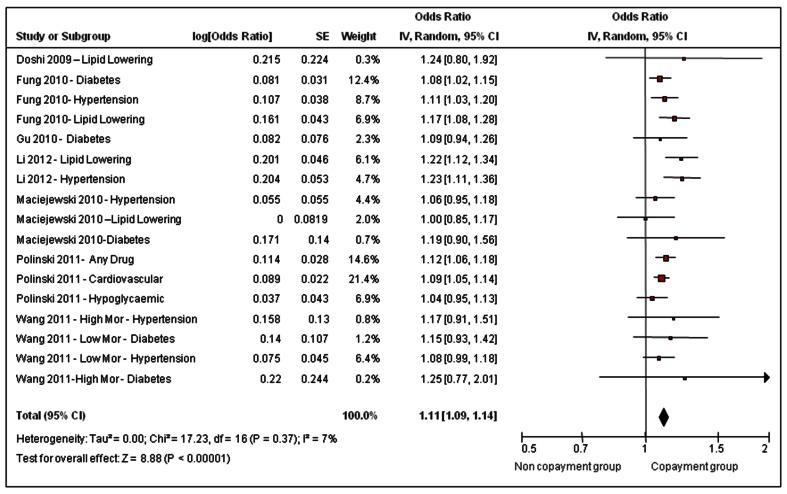
Effect of requirement to pay a copayment for prescription drugs on non-adherence in a publicly insured population.

The meta-analysis included 199, 996 people overall; 74,236 people in the copayment group and 125, 760 people in the non-copayment group. These numbers may overestimate the true number of individuals because the 7 included studies contributed 16 separate patient groups, and there may have been some overlap. **Figure 2** shows the results of the meta-analysis which plots the outcome, non-adherence, as affected by the exposure, requirement to copay for prescription drugs. The summary odds ratio for non-adherence is 1.11 (95% CI 1.09–1.14; P = <0.00001) in the copayment group. Results were consistent across studies; an acceptable level of heterogeneity at I^2^ = 7%, (p = 0.37) was observed.

## Discussion

This meta-analysis has found an 11% increase in odds of non-adherence when publicly insured patients are required to copay for their prescription medicines. This is a pertinent result because the question regarding adherence to medicines in a cost sharing environment was still inconclusively quantitatively answered by prior reviews [18], [33]–[35].

Medication classes that appeared more than once in the meta-analysis included those for hypertension, hyperlipidaemia and diabetes; medicines which are regarded as being essential. This gives this meta-analysis particular relevance; because lack of adherence to these medicines can be important clinically and economically.

While adherence is a surrogate outcome for clinical outcomes, there is a body of literature which allows extrapolation of this finding to give meaningful clinical results. People with diabetes are traditionally poor adherers to chronic medications with reported levels of adherence as low as 50% [53]. This occurs despite the knowledge that tight glycaemic control results in fewer complications for patients and also has economic benefits [31], [54]–[56]. Similarly, rates of adherence to hypertensive medicines are widely reported to be poor; with rates of adherence at 50% 1 year after starting treatment [57]. Well-controlled hypertension is seen at levels of adherence at over 80% [58]. Cherry *et al* [59] showed approximately a double relative risk of myocardial infarction, stroke and angina in non-adherers verses those who have “ideal” adherence to anti-hypertensive medicines. Their research also outlined the economic burden of non-adherence, costing roughly $8,500/life year gained more than full adherence. It has been found that non-adherence to oral hypoglycaemics, anti-hypertensives and statins in more than 11,000 patients with diabetes was significantly associated with all cause hospitalisations and all cause mortality [60]. These results have financial implications as described by Sokol *et al*, who demonstrated that adherence to medicines used to treat diabetes and hypercholesteremia reduces expenditure in overall health costs [27]. In hypertensive disease, the same results have been shown by McCombs *et al* [61]. The result of this meta-analysis shows copayment to be an additional risk factor for non-adherence. Given the already low adherence profiles to these essential drugs and the associated costly repercussions, an 11% increased odds of non-adherence to these medicines may be important clinically and economically.

The results of this meta-analysis agree with the qualitative results of reviews that have been published in the broader area of cost-sharing and utilisation of drugs [33]–[35]. Previous reviews quantified broadly defined utilisation by calculating elasticities [18], [32], [34]. Nevertheless, this is the first review to encompass a meta-analysis and give a numerical summary measure of the odds of non-adherence when individuals are required to copay for medicines. In addition, this review builds upon recent related reviews such as Eaddy *et al* [32] by focusing solely on publicly insured populations.

Pharmaceutical expenditure is difficult to contain at present due to a global aging population and the increased incidence of morbidity that this is associated with. To compound this, the growth of the pharmaceutical bio-technology industry and the development of biological drugs that are increasingly prescribed will serve to maintain, if not increase, public spending on pharmaceuticals. It is imperative for policy makers and health economists to devise practical and balanced cost sharing policies that do not represent a barrier to cheap, effective medicines which produce health gains at a large population level. Such health gains result in large financial savings by maintaining public health and by decreased health services utilisation [27], [62]. Simultaneously however, drug expenditure policies must account for moral hazard and attempt to confer upon the patient the notion of cost responsibility. Due to clinical, financial and political influences cost-sharing policies are often difficult to formulate. This meta-analysis will contribute to the body of evidence that should be used as a guide in future decision making.

### Limitations

Despite strict inclusion and exclusion criteria which were developed to obtain studies that would be comparable, some differences in research methodologies persisted. First, follow up times in the included studies varied widely, ranging from 3 months to 2 years. Secondly, this type of research is vulnerable to confounding and not all studies controlled for the same confounders. For example 2 studies attempted to control for an introduced fee for physician care [49], [50], whereas another paper experiences the same physician fee, but does not control for this confounder [48]. Thirdly, the included studies were of weak to moderate quality. Given the non-experimental nature of this research this is a problem that is difficult to avoid. Regardless, the quality of included studies should be borne in mind when interpreting the summary effect measure. There are methodological strengths in the included studies such as propensity score matching that attenuate the biased nature of some observational research designs. However propensity score matching can leave residual confounding between groups and cannot account for unknown or immeasurable confounding as a randomised controlled trial only can.

It may be worth noting that qualitative analysis of interrupted time series studies [5] may provide an interesting insight into patient behaviours over a period of time. Analysis of such results would show how adherence fluctuates in the months preceding the policy change, for example, adherence to β-blockers introduced after a myocardial infarction can fall rapidly even before a copay policy change [5]. However, a meta-analysis of such data would be impractical, thus the method employed in the meta-analysis presented here is a pragmatic way of analysing the key research question.

Next, the studies included in this review were concerned with drugs that act primarily in cardiovascular disease and diabetes. Therefore the results of the meta-analysis may not be extrapolated to other disease groups such as cancer or pain. However, given that cardiovascular disease, ischaemic heart disease and diabetes are included in the top ten causes of death in high income countries [63] these results still have high relevancy and a wide generalisabilty. Despite this, external validity may suffer as some studies excluded the poorest members of society due to different coverage status for these people [46], [47]. Furthermore, the studies included in this review focus on elderly populations. Therefore, the results may not be applicable to younger vulnerable populations. However, given that elderly populations are the biggest users of pharmaceuticals [64], [65], this review still gives pertinent information. Further analysis in younger, low income populations should be undertaken to fill in the information gaps.

There may have been a degree of publication bias present. Efforts were made to overcome this problem in the development of the search strategy which encompassed 8 electronic databases, the grey literature and hand-searching. The potential presence of publication bias should be kept in mind when interpreting the summary effect of this meta-analysis.

Lastly, this meta-analysis was explicit in the intervention analysed i.e., an introduced or increased copayment. Results from this analysis may not be extendable to other cost-sharing policies such as co-insurance, because the effects of different cost-sharing policies are not necessarily always comparable [4]. However, there is no reason why these results cannot be extrapolated in a directional manner for other policies.

## Conclusions

This meta-analysis showed an 11% increased odds of non-adherence to medicines in publicly insured populations involved in a system where copayments for medicines are required. Reductions in adherence to medications, especially essential medicines, can be detrimental to health status and causes increases in expenditure via hospital admissions. Hence, the results of this meta-analysis should be taken into account at a policy and health systems level to aid in striking a balance between the financial benefits and financial repercussions of cost-sharing policies.

## Supporting Information

Figure S1 **Sensitivity analysis for study design; CBA studies.**
(TIF)

Figure S2 **Sensitivity analysis for study design; Cohort studies.**
(TIF)

Figure S3 **Sensitivity analysis for gender differences in studies; Studies with mixed genders.**
(TIF)

Figure S4 **Sensitivity analysis for gender differences in studies; Studies with predominant male distribution.**
(TIF)

Table S1 **Excluded studies and reasons for exclusion.**
(DOCX)
