# Supplementary figures and images for: The Effect of Copayments for Prescriptions on Adherence to Prescription Medicines in Publicly Insured Populations; A Systematic Review and Meta-Analysis
Source: PLoS One. 2013 May 28;8(5):e64914. doi: 10.1371/journal.pone.0064914 (PMC3665806; doi:10.1371/journal.pone.0064914)

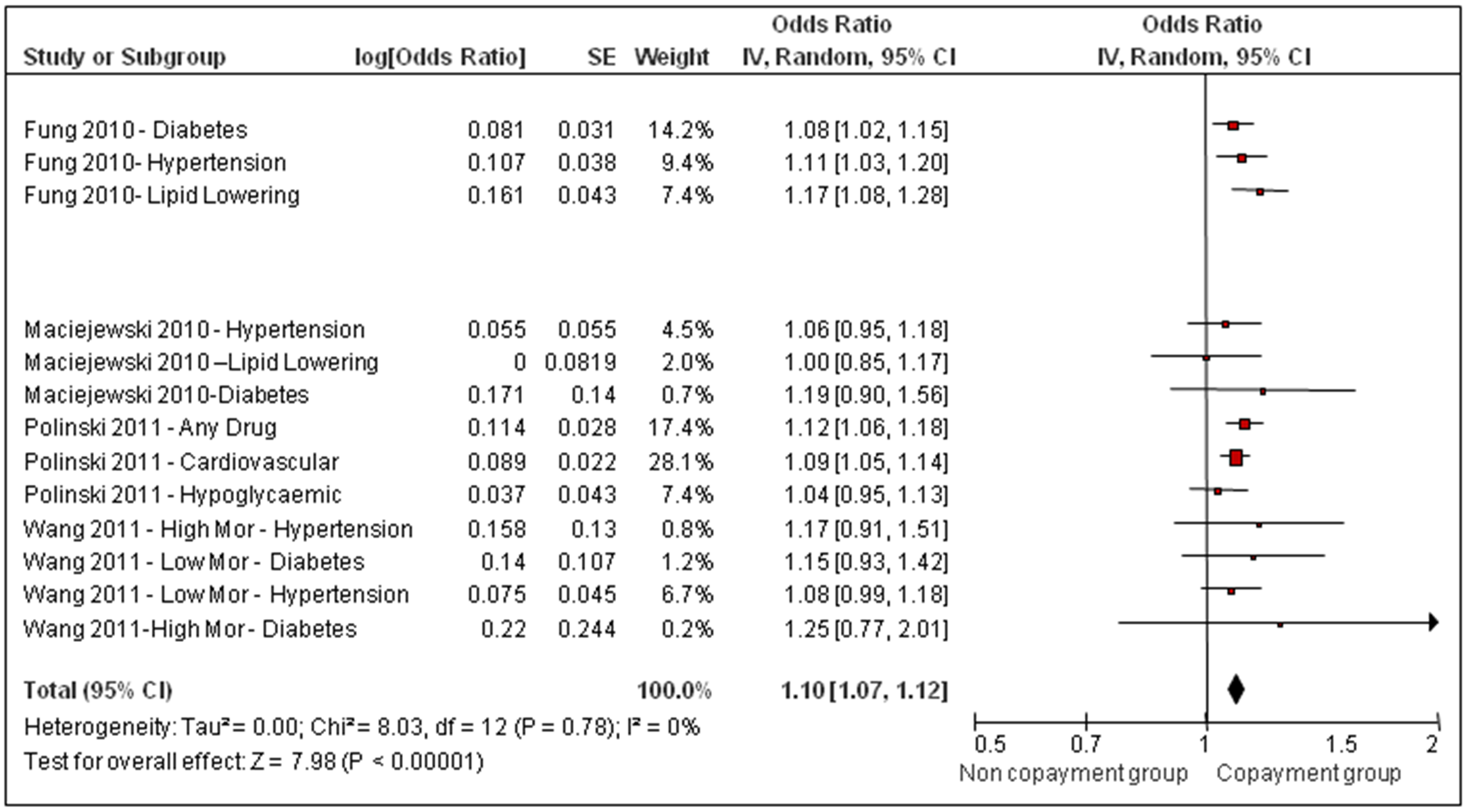

Supplement: Figure S1 — Sensitivity analysis for study design; CBA studies. (TIF) [file pone.0064914.s001.tif]

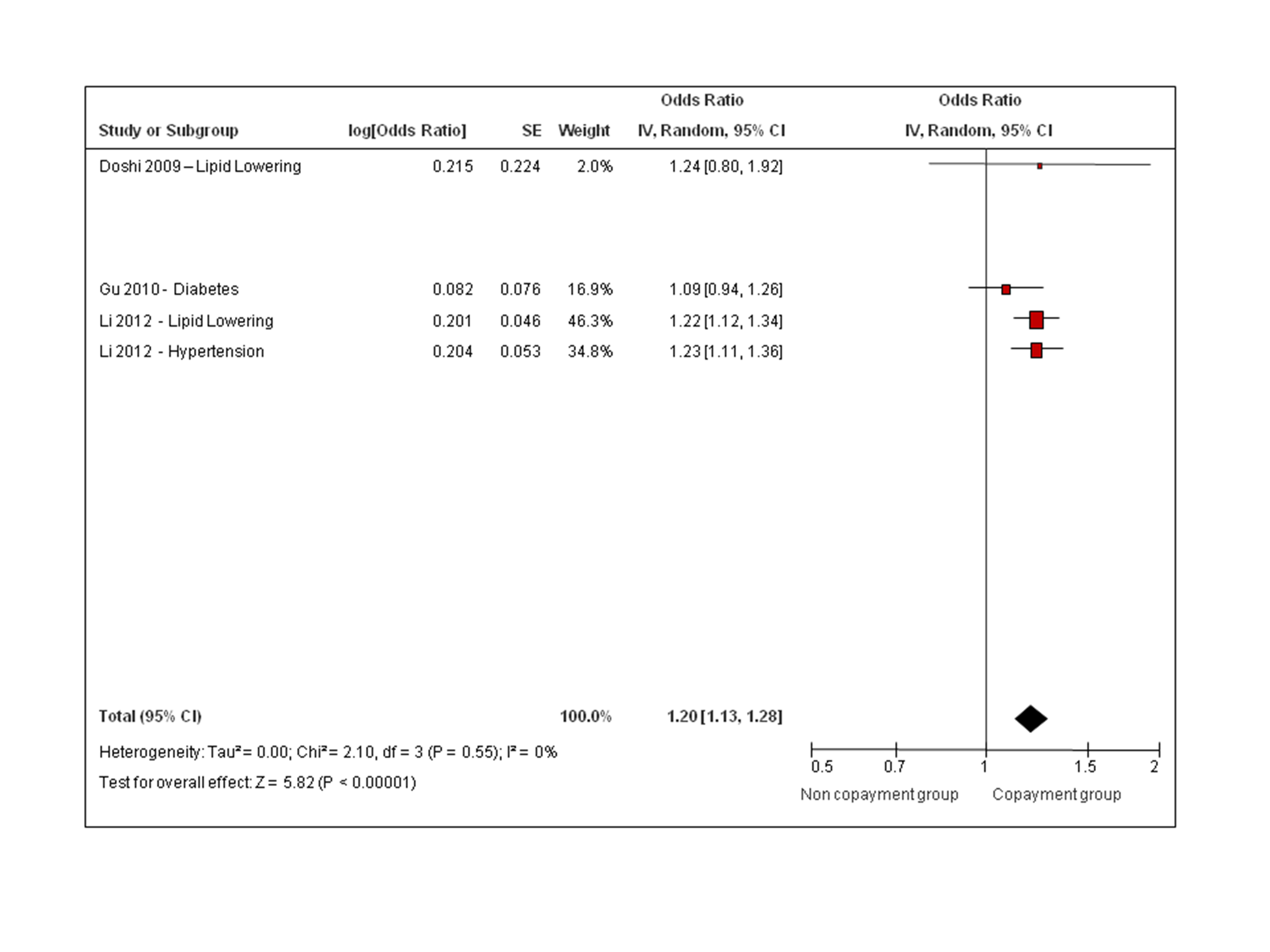

Supplement: Figure S2 — Sensitivity analysis for study design; Cohort studies. (TIF) [file pone.0064914.s002.tif]

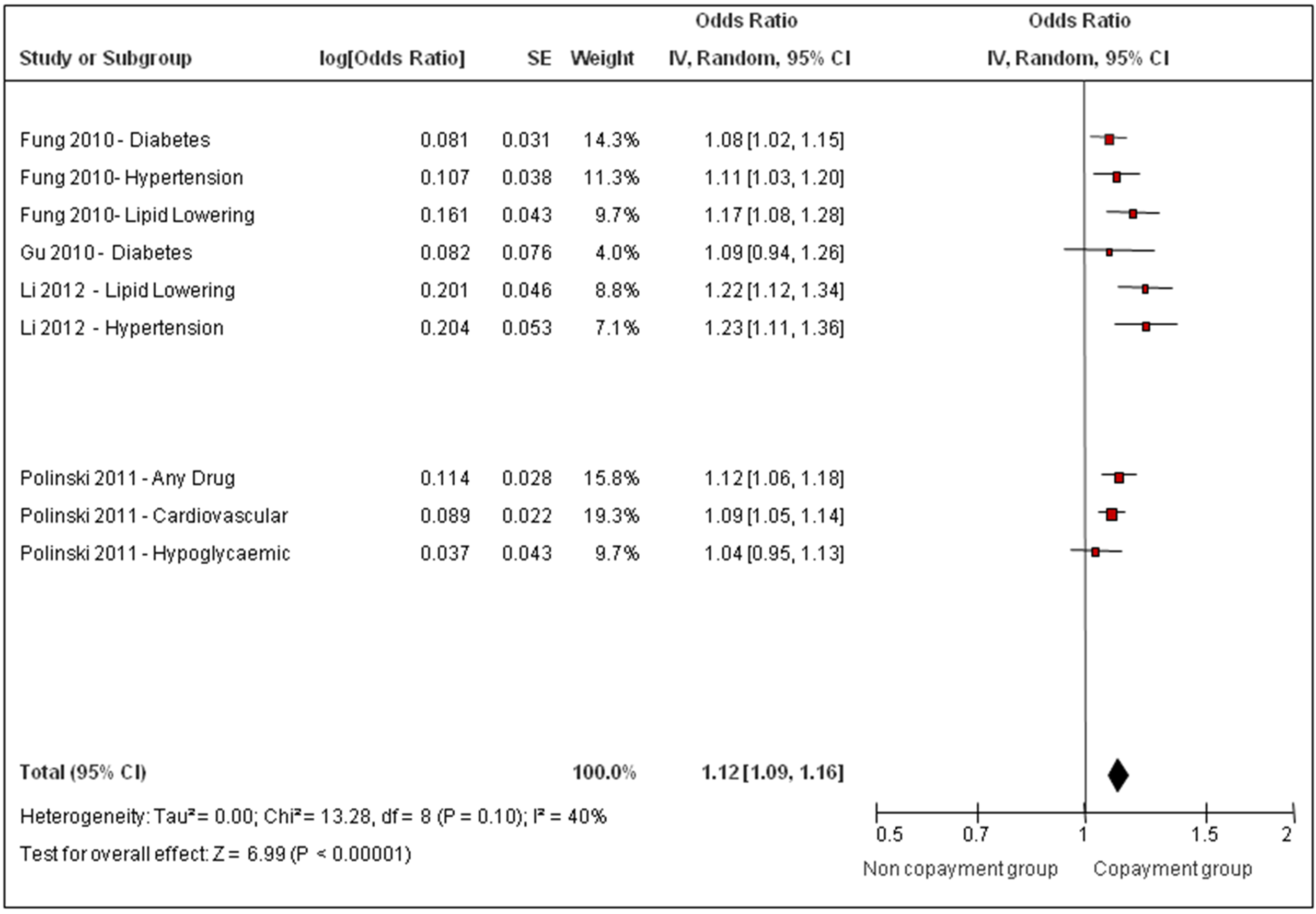

Supplement: Figure S3 — Sensitivity analysis for gender differences in studies; Studies with mixed genders. (TIF) [file pone.0064914.s003.tif]

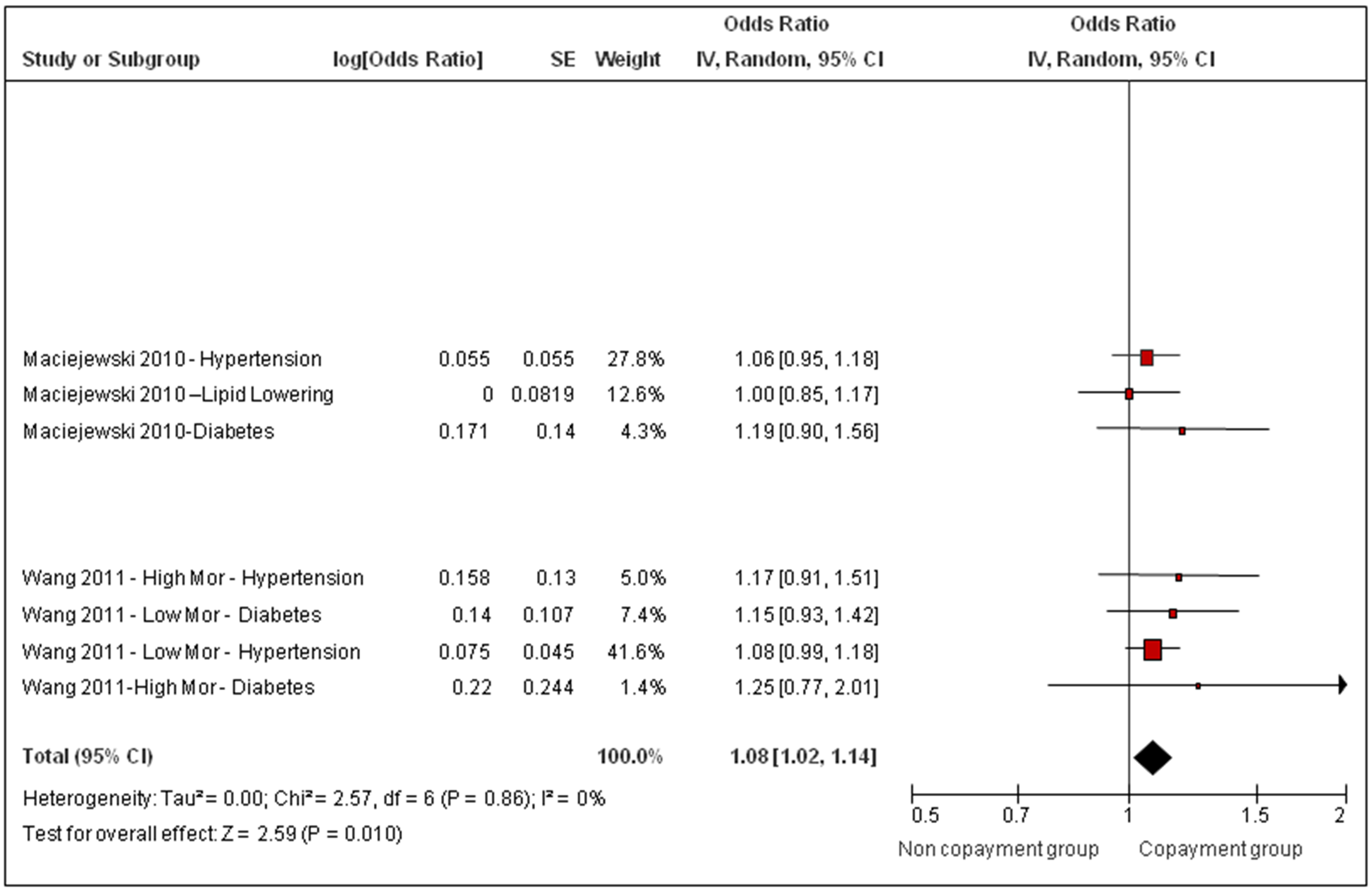

Supplement: Figure S4 — Sensitivity analysis for gender differences in studies; Studies with predominant male distribution. (TIF) [file pone.0064914.s004.tif]
