# Supplementary material for: The Effect of Copayments for Prescriptions on Adherence to Prescription Medicines in Publicly Insured Populations; A Systematic Review and Meta-Analysis
Source: PLoS One. 2013 May 28;8(5):e64914. doi: 10.1371/journal.pone.0064914 (PMC3665806; doi:10.1371/journal.pone.0064914)
Supplement: Table S1 — Excluded studies and reasons for exclusion. (DOCX) [file pone.0064914.s005.docx]

| **Excluded papers and reasons for exclusion in original search carried out between November and December 2011** | |
| --- | --- |
| Schneeweiss et al, 2007 [[1](#_ENREF_1)] (beta blockers) | Could not retrieve adjusted estimates |
| Schneeweiss et al, 2007 [[2](#_ENREF_2)] (statins) | Could not retrieve adjusted estimates |
| Farley et al, 2010 [[3](#_ENREF_3)] | Could not retrieve data for meta-analysis |
| Hsu et al, 2006 [[4](#_ENREF_4)] | Could not retrieve data for meta-analysis |
| Domino et al, 2011 [[5](#_ENREF_5)] | Could not retrieve data for meta-analysis |
| Dormuth et al, 2006 [[6](#_ENREF_6)] | Does not study adherence – uses DDD but in a utilisation capacity |
| Hynd et al, 2009 [[7](#_ENREF_7)] | Does not study adherence – uses DDD but in a utilisation capacity |
| Raebel et al, 2008 [[8](#_ENREF_8)] | Use MRA – Medication Refill Adherence not one of the specified outcome measures |
| Starmans et al, 1994 [[9](#_ENREF_9)] | Does not study adherence – uses DDD but in a utilisation capacity |
| Taira et al, 2006 [[10](#_ENREF_10)] | Cross sectional  No control group; comparison group is private  No data on medicare baseline characteristics (all groups characterised together) |
| Ye etal, 2007 [[11](#_ENREF_11)] | Medicare and commercial data were combined for analysis |
| Zeng et al, 2010 [[12](#_ENREF_12)] | Models effect of copayment on adherence however this modelling isn’t broken down by medicare/commercial – just aggregate copayment levels. |
| Cole et al, 2006 [[13](#_ENREF_13)] | Medicare supplemental and commercial mixed together in results |
| Neugut et al, 2011 [[14](#_ENREF_14)] | Reports 8.9% non-adherence in our medicare sample over 2 years, this isn’t compared to anything else. Also this is for varying copays. |
| Foster et al, 2011 [[15](#_ENREF_15)] | No appropriate comparison or control group |
| Ong et al, 2003 [[16](#_ENREF_16)] | Does not study adherence – uses DDD but in a utilisation capacity |

| **Excluded papers and reasons for exclusion in updated search carried out in September 2012** | |
| --- | --- |
| Assayag et al, 2011 [[17](#_ENREF_17)] | Comparison group is a private population |
| Zeng et al 2012 [[18](#_ENREF_18)] | Commercially insured |
| Wladysiuk et al, 2011 [[19](#_ENREF_19)] | Uses DDDs |
| Lee et al, 2012 [[20](#_ENREF_20)] | Does not look at adherence. Looks at utilisation. |
| Lau et al, 2011 [[21](#_ENREF_21)] | Review article |
| Kleinman et al, 2012 [[22](#_ENREF_22)] | Mixed populations |
| Kim et al, 2011 [[23](#_ENREF_23)] | Medicare and Medicaid patients were excluded |
| Kibicho and Pinkerton, 2012 [[24](#_ENREF_24)] | Analyses health policies – doesn’t look at adherence. |
| Kennedy et al, 2011 [[25](#_ENREF_25)] | Does not assess adherence |
| Geers et al, 2011 [[26](#_ENREF_26)] | Does not look at copay |
| Einecke, 2011 [[27](#_ENREF_27)] | Review/comment article |
| Burke, 2012 [[28](#_ENREF_28)] | Review article |
| Briesacher et al, 2011 [[29](#_ENREF_29)] | Self-reported data |
| APhA Abstracts, 2011 [[30](#_ENREF_30)] | Abstracts – none relevant |
| Mahabaleshwarkar [[31](#_ENREF_31)] | Contacted in march. They did not publish the work. |
| Palmer et al, 2011[[32](#_ENREF_32)] | Contacted authors not published until October |
| Subramanian, 2011 [[33](#_ENREF_33)] | No PDC/ReCOMP/MPR |
| Fung et al, 2012 [[34](#_ENREF_34)] | Contacted authors – no reply |

1. Schneeweiss S, Patrick AR, Maclure M, Dormuth CR, Glynn RJ (2007) Adherence to beta-blocker therapy under drug cost-sharing in patients with and without acute myocardial infarction. American Journal of Managed Care 13: 445-452.

2. Schneeweiss S, Patrick AR, Maclure M, Dormuth CR, Glynn RJ (2007) Adherence to statin therapy under drug cost sharing in patients with and without acute myocardial infarction: A population-based natural experiment. Circulation 115: 2128-2135.

3. Farley JF (2010) Medicaid prescription cost containment and schizophrenia: A retrospective examination. Medical Care 48: 440-447.

4. Hsu J, Price M, Huang J, Brand R, Fung V, et al. (2006) Unintended consequences of caps on Medicare drug benefits. N Engl J Med 354: 2349-2359.

5. Domino ME, Martin BC, Wiley-Exley E, Richards S, Henson A, et al. (2011) Increasing time costs and copayments for prescription drugs: An analysis of policy changes in a complex environment. Health Services Research 46: 900-919.

6. Dormuth CR, Glynn RJ, Neumann P, Maclure M, Brookhart AM, et al. (2006) Impact of two sequential drug cost-sharing policies on the use of inhaled medications in older patients with chronic obstructive pulmonary disease or asthma. Clinical Therapeutics 28: 964-978.

7. Hynd A, Roughead EE, Preen DB, Glover J, Bulsara M, et al. (2009) Increased patient co-payments and changes in PBS-subsidised prescription medicines dispensed in Western Australia. Australian & New Zealand Journal of Public Health 33: 246-252.

8. Raebel MA, Delate T, Ellis JL, Bayliss EA (2008) Effects of reaching the drug benefit threshold on medicare members? Healthcare utilization during the first year of medicare part D. Medical Care 46: 1116-1122.

9. Starmans B, Janssen R, Schepers M, Verkooijen M (1994) The effect of a patient charge and a prescription regulation on the use of antihypertension drugs in Limburg, The Netherlands. Health Policy 26: 191-206.

10. Taira DA, Wong KS, Frech-Tamas F, Chung RS (2006) Copayment level and compliance with antihypertensive medication: Analysis and policy implications for managed care. American Journal of Managed Care 12: 678-683.

11. Ye X, Gross CR, Schommer J, Cline R, St Peter WL (2007) Association between copayment and adherence to statin treatment initiated after coronary heart disease hospitalization: a longitudinal, retrospective, cohort study. Clinical Therapeutics 29: 2748-2757.

12. Zeng F, Patel BV, Andrews L, Frech-Tamas F, Rudolph AE (2010) Adherence and persistence of single-pill ARB/CCB combination therapy compared to multiple-pill ARB/CCB regimens. Current Medical Research & Opinion 26: 2877-2887.

13. Cole JA, Norman H, Weatherby LB, Walker AM (2006) Drug copayment and adherence in chronic heart failure: Effect on cost and outcomes. Pharmacotherapy 26: 1157-1164.

14. Neugut AI, Subar M, Wilde ET, Stratton S, Brouse CH, et al. (2011) Association between prescription co-payment amount and compliance with adjuvant hormonal therapy in women with early-stage breast cancer. Journal of Clinical Oncology 29: 2534-2542.

15. Foster SA, Foley KA, Meadows ES, Johnston JA, Wang SS, et al. (2011) Adherence and persistence with teriparatide among patients with commercial, Medicare, and Medicaid insurance. Osteoporos Int 22: 551-557.

16. Ong M, Catalano R, Hartig T (2003) A time-series analysis of the effect of increased copayments on the prescription of antidepressants, anxiolytics, and sedatives in Sweden from 1990 to 1999. Clinical Therapeutics 25: 1262-1275.

17. Assayag J, Forget A, Kettani FZ, Beauchesne MF, Blais L (2011) Impact of type of drug insurance on adherence and costs of antidepressant drugs. Journal of Population Therapeutics and Clinical Pharmacology 18: e276.

18. Zeng F, Knoth RL, Patel BV, Kim E, Tran QV, et al. (2012) Impact of health plan restrictions on antipsychotic medication adherence and persistence. American Journal of Pharmacy Benefits 4: e22-e31.

19. Wladysiuk M, Araszkiewicz A, Godman B, Szabert K, Barbui C, et al. (2011) Influence of Patient Co-payments on Atypical Antipsychotic Choice in Poland: Implications Once Generic Atypicals Are Available. Applied Health Economics and Health Policy 9: 101-110.

20. Lee IH, Bloor K, Hewitt C, Maynard A (2012) The effects of new pricing and copayment schemes for pharmaceuticals in South Korea. Health Policy 104: 40-49.

21. Lau DT, Briesacher BA, Touchette DR, Stubbings J, Ng JH (2011) Medicare Part D and Quality of Prescription Medication Use in Older Adults. Drugs & Aging 28: 797-807.

22. Kleinman NL, Sadosky A, Seid J, Martin RC, Labiner DM (2012) Costs, work absence, and adherence in patients with partial onset seizures prescribed gabapentin or pregabalin. Epilepsy Research.

23. Kim YA, Rascati KL, Prasla K, Godley P, Goel N, et al. (2011) Retrospective evaluation of the impact of copayment increases for specialty medications on adherence and persistence in an integrated health maintenance organization system. Clinical Therapeutics 33: 598-607.

24. Kibicho J, Pinkerton SD (2012) Multiple Drug Cost Containment Policies In Michigan's Medicaid Program Saved Money Overall, Although Some Increased Costs. Health Affairs 31: 816-826.

25. Kennedy J, Dipzinski A, Roll J, Coyne J, Blodgett E (2011) Medicare prescription drug plan coverage of pharmacotherapies for opioid and alcohol dependence in WA. Drug & Alcohol Dependence 114: 201-206.

26. Geers HC, Bouvy ML, Heerdink ER (2011) Influence of therapeutic complexity on medication adherence in the Netherlands. Archives of Internal Medicine 171: 864-865; author reply 865.

27. Einecke D (2011) Secondary prevention. Pills without co-pay reduce complications after myocardial infarct. MMW Fortschritte der Medizin 153: 15-15.

28. Burke M (2012) DO HIGHER OUT-OF-POCKET COSTS REDUCE THE USE OF SOME MEDICATIONS? Contemporary Pediatrics 29: 13-13.

29. Briesacher BA, Zhao Y, Madden JM, Zhang F, Adams AS, et al. (2011) Medicare part D and changes in prescription drug use and cost burden: national estimates for the Medicare population, 2000 to 2007. Medical Care 49: 834-841.

30. (2011) APhA2011 Abstracts. Journal of the American Pharmacists Association 51.

31. Mahabaleshwarkar R, Banahan BFI (2011) Effect of medicare part d coverage GAP on medication consumption behaviors: Case of oral anti-diabetic medications. Value in Health 14: A102.

32. Palmer LA, Fowler R, Shi N, Dastani H, Abouzaid S, et al. (2011) Impact of patient cost-sharing arrangements for disease modifying therapies on treatment compliance among patients with multiple sclerosis in the United States. Value in Health 14: A328-A329.

33. Subramanian S (2011) Impact of Medicaid copayments on patients with cancer: lessons for Medicaid expansion under health reform. Medical Care 49: 842-847.

34. Fung V, Price M, Busch A, Dow W, Fireman B, et al. (2012) CB7-02: Medicare Part D Cost-sharing and Antipsychotic Drug Use in Two Medicare Advantage Systems. Clin Med Res 10: 182.
